# Differences of survival benefits brought by various treatments in ovarian cancer patients with different tumor stages

**DOI:** 10.1186/s13048-023-01173-7

**Published:** 2023-05-11

**Authors:** Tingshan He, Hong Li, Zhiqiao Zhang

**Affiliations:** Department of Infectious Diseases, Shunde Hospital, Southern Medical University, Guangdong, 528303 Shunde China

**Keywords:** Ovary cancer, Restricted mean survival time, Precision medicine, Prognostic model, Artificial intelligence, Individual mortality risk prediction

## Abstract

**Purpose:**

The current study aimed to explore the prognosis of ovarian cancer patients in different subgroup using three prognostic research indexes. The current study aimed to build a prognostic model for ovarian cancer patients.

**Methods:**

The study dataset was downloaded from Surveillance Epidemiology and End Results database. Accelerated Failure Time algorithm was used to construct a prognostic model for ovary cancer.

**Results:**

The mortality rate in the model group was 51.6% (9,314/18,056), while the mortality rate in the validation group was 52.1% (6,358/12,199). The current study constructed a prognostic model for ovarian cancer patients. The C indexes were 0.741 (95% confidence interval: 0.731–0.751) in model dataset and 0.738 (95% confidence interval: 0.726–0.750) in validation dataset. Brier score was 0.179 for model dataset and validation dataset. The C indexes were 0.741 (95% confidence interval: 0.733–0.749) in bootstrap internal validation dataset. Brier score was 0.178 for bootstrap internal validation dataset.

**Conclusion:**

The current research indicated that there were significant differences in the survival benefits of treatments among ovarian cancer patients with different stages. The current research developed an individual mortality risk predictive system that could provide valuable predictive information for ovarian cancer patients.

**Graphical Abstract:**

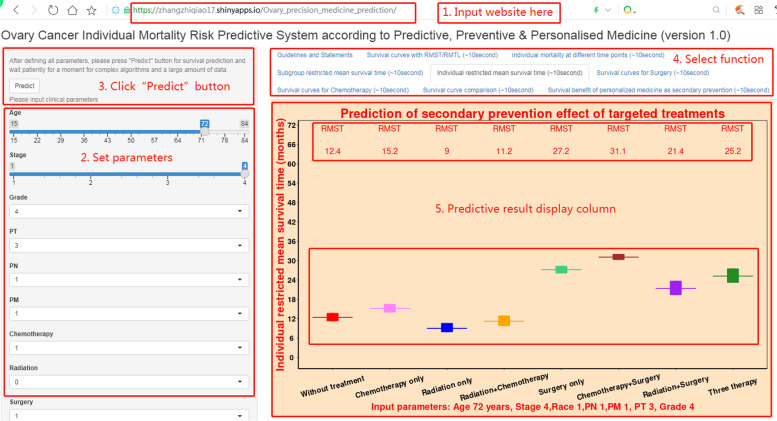

**Supplementary Information:**

The online version contains supplementary material available at 10.1186/s13048-023-01173-7.

## Introduction

In 2020, ovarian cancer caused about 310,000 new cases and about 200,000 deaths worldwide [1]. Eighty percent of ovarian cancer patients were in advanced stage for initial diagnosis [2]. The 5-year survival rate of ovarian cancer patients with metastasis was about 10%-20% [3]. The 5-year survival rates of patients with stage I, II, III, and IV ovarian cancer were 89%, 70%, 36%, and 17%, whereas the 10-year survival rates were 84%, 59%, 23%, and 8%, respectively [4]. The 8-year survival rates of patients with stage I/II and stage III/IV ovarian cancer were 85.7% and 20.0%, respectively [5]. The 5‐year survival rate of advanced stage ovarian cancer was reported to be 18%-30% [6]. Early identification of patients with high mortality risk and individualized treatment were of great significance in improving the prognosis of ovarian cancer patients.

At present, several prognostic nomograms were constructed to predict the prognosis of ovarian cancer patients [7–10]. However, for ovarian cancer patients, the individual predicted survival curve in the whole follow-up cycle was more clinically valuable than the predicted survival rate at a single time point. Furthermore, clinical patients might be more concerned about the individual predicted survival time, which was easier to understand and compare.

The restricted mean survival time (RMST) is the sum of the areas under the survival curve within a specific time range [11–15]. Restricted mean survival time has been widely used in different clinical research [11–15]. The current study will demonstrate and compare the survival benefits of different treatments through restricted mean survival time.

Different from the traditional reactive treatment model, Predictive, Preventive and Personalized Medicine (PPPM) model pays more attention to medical prediction, targeted intervention and personalised medical services [16]. PPPM model has been widely used in the prevention, control and management of different diseases [17–20]. The emergence of medical big data provides rich materials for discovering new prevention methods, optimizing treatment effects and promoting personalized medicine [21]. However, there is no clinical study on PPPM strategy in ovarian cancer patients.

The current study aimed to explore the prognosis of ovarian cancer patients in different subgroup using three prognostic research indexes. Furthermore, the current study aimed to build a prognostic model for ovarian cancer patients.

## Method

### Study dataset

We searched Surveillance Epidemiology and End Results (SEER) database from January 2004 to December 2015. Seven retrieval strategies used in the current study was presented in the Supplementary document 1. According to these search conditions, 39,155 eligible patients with complete pathological information and survival information were included in the current study. The pathological staging and grading diagnostic criteria of all ovarian cancer refered to the recommendations of American Joint Committee on Cancer (AJCC) [22–24]. The following patients were excluded from the current study due to lack of the following information: treatment (*n* = 91), grade (*n* = 8,724), and race (*n* = 85). Finally, 30,255 patients were enrolled in the final survival analysis. The patient enrollment flow chart was presented in the Supplementary document 2. Supplementary document 3 presented comparison analysis results between survival cohort and died cohort.

### Variable selection and model development

The original dataset was divided into modeling dataset and validation dataset by random method. Multivariable Cox proportional risk regression algorithm was used to identify potential markers of ovarian cancer prognostic model. The accelerated failure time (AFT) algorithm was used to develop prognostic model for ovarian cancer patients. The C indexes and Brier score were used to assess the predictive performance of prognostic model. On the premise that effective external verification queue research can’t be obtained, the internal verification research based on boortrap resampling dataset was recommended as prerequisite for predictive model development by Transparent Reporting of a multivariable prediction model for Individual Prognosis Or Diagnosis [25, 26]. The current study performed internal validation based on dataset resampled through boortrap resampling method.

### Statistical analysis

The current study used R language 4.0.5 (R Project, Vienna, Austria) for statistical analysis. Prognostic model was developed using accelerated failure time (AFT) algorithm [27–29]. Restricted mean survival time (RMST) was calculated with following formula [30]: $\mathrm{RMST }={\sum }_{0}^{t}\mathrm{S}\left(\mathrm{t}\right)\mathrm{D}\left(\mathrm{t}\right)$. Restricted mean time lost (RMTL) was calculated with following formula [30]: $\mathrm{RMTL }={\sum }_{0}^{t}{\left[1-\mathrm{S}\left(\mathrm{t}\right)\right]}^{*}\mathrm{D}\left(\mathrm{t}\right)$.

## Results

### Clinical characteristics

The enrolled patients were randomly assigned to model subgroup (*n* = 18,056) and validation subgroup (12,199) according to the proportion of 6/4. The mortality rate in the model group was 51.6% (9,314/18,056), while the mortality rate in the validation group was 52.1% (6,358/12,199). There was no significant difference in baseline characteristics between model subgroup and validation subgroup after randomization (Table 1).

**Table 1:** Comparison of baseline characteristics of model cohort and validation cohort

Parameter	Stratification	Total	Model dataset	Validation dataset	Test_value	*P*_value
Survival_status[n(%)]	Survival	14,583	8742(28.89)	5841(19.31)	0.81	0.367
	Dead	15,672	9314(30.78)	6358(21.01)		
Stage[n(%)]	Stage I	8583	5091(16.83)	3492(11.54)	0.92	0.821
	Stage II	3325	1999(6.61)	1326(4.38)		
	Stage III	12,940	7724(25.53)	5216(17.24)		
	Stage IV	5407	3242(10.72)	2165(7.16)		
PT[n(%)]	T1	9128	5411(17.88)	3717(12.29)	0.95	0.621
	T2	4387	2634(8.71)	1753(5.79)		
	T3	16,740	10,011(33.09)	6729(22.24)		
PN[n(%)]	No	23,238	13,861(45.81)	9377(30.99)	0.04	0.85
	Yes	7017	4195(13.87)	2822(9.33)		
PM[n(%)]	No	24,848	14,814(48.96)	10,034(33.16)	0.2	0.654
	Yes	5407	3242(10.72)	2165(7.16)		
Radiation[n(%)]	No	29,771	17,762(58.71)	12,009(39.69)	0.19	0.664
	Yes	484	294(0.97)	190(0.63)		
Chemotherapy[n(%)]	No	7009	4145(13.7)	2864(9.47)	1.08	0.298
	Yes	23,246	13,911(45.98)	9335(30.85)		
Surgery[n(%)]	No	567	355(1.17)	212(0.7)	1.94	0.164
	Yes	29,688	17,701(58.51)	11,987(39.62)		
Grade[n(%)]	Moderately differentiated	3527	2131(7.04)	1396(4.61)	5.34	0.148
	Poorly differentiated	5752	3359(11.1)	2393(7.91)		
	Undifferentiated	13,612	8141(26.91)	5471(18.08)		
	Well differentiated	7364	4425(14.63)	2939(9.71)		
Race[n(%)]	White	25,328	15,086(49.86)	10,242(33.85)	1.62	0.656
	Black	1893	1128(3.73)	765(2.53)		
	Asian or Pacific Islander	2814	1706(5.64)	1108(3.66)		
	American Indian/Alaska Native	220	136(0.45)	84(0.28)		
Laterality[n(%)]	Left	8538	5131(16.96)	3407(11.26)	1.81	0.405
	Right	8538	5047(16.68)	3491(11.54)		
	Both	13,179	7878(26.04)	5301(17.52)		
Schedule[n(%)]	Without Treatment	146	104(0.34)	42(0.14)	14.06	0.05
	Chemotherapy only	406	241(0.8)	165(0.55)		
	Radiation only	5	2(0.01)	3(0.01)		
	Radiation + Chemotherapy	10	8(0.03)	2(0.01)		
	Surgery only	6803	4010(13.25)	2793(9.23)		
	Chemotherapy + Surgery	22,416	13,407(44.31)	9009(29.78)		
	Radiation + Surgery	55	29(0.1)	26(0.09)		
	Three therapy	414	255(0.84)	159(0.53)		
Survival_month [month]^a^		60.0(29.0,103.0)	59.0(29.0,101.0)	60.0(29.0,105.0)	108,724,809.5	0.059
Age [year]^a^		58.0(50.0,67.0)	58.0(50.0,67.0)	58.0(50.0,67.0)	110,041,017	0.902

^a^Kruskal-Wallis Test; other: Chi square test

### Prognostic analysis at overall level

**Fig. 1:**
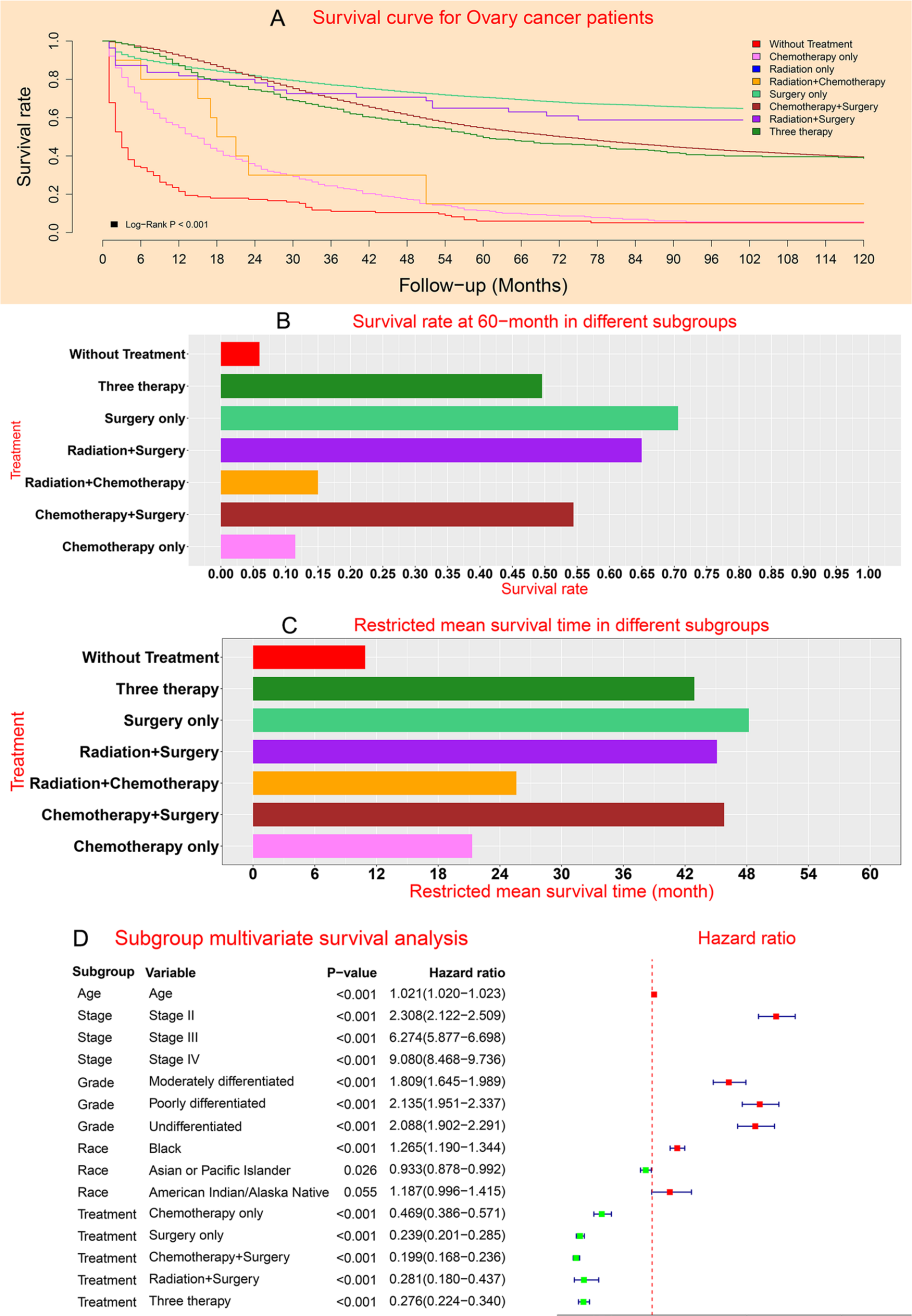
Prognostic performance of patients in different stages: **A** Survival curve; **B** Survival rate; **C** Restricted mean survival time; **D** Multivariable forest chart

Survival curve analysis showed that the survival of without treatment subgroup and chemotherapy only subgroup were the worst two, while the survival of surgery only subgroup and radiation plus surgery subgroup were the best two (Fig. 1A). The 60-month survival rate analysis demonstrated that the survival rate of without treatment subgroup and chemotherapy only subgroup were the worst two, while the survival of surgery only subgroup and radiation plus surgery subgroup were the best two (Fig. 1B). The Restricted mean survival time analysis demonstrated that the survival rate of without treatment subgroup and chemotherapy only subgroup were the worst two, while the survival of surgery only subgroup and chemotherapy plus surgery subgroup were the best two (Fig. 1C). Multivariate Cox survival analysis showed that the prognosis of chemotherapy only subgroup, surgery only subgroup, chemotherapy plus surgery subgroup, radiation plus surgery subgroup, and three therapy subgroup were significantly better than that of without treatment subgroup (Fig. 1D). In multivariate analysis, the reference baseline subgroup was defined as white subgroup for race, well differentiated subgroup for grade, without treatment subgroup for treatment, Stage I subgroup for pathological stage.

### Survival analysis at subgroup level

**Fig. 2:**
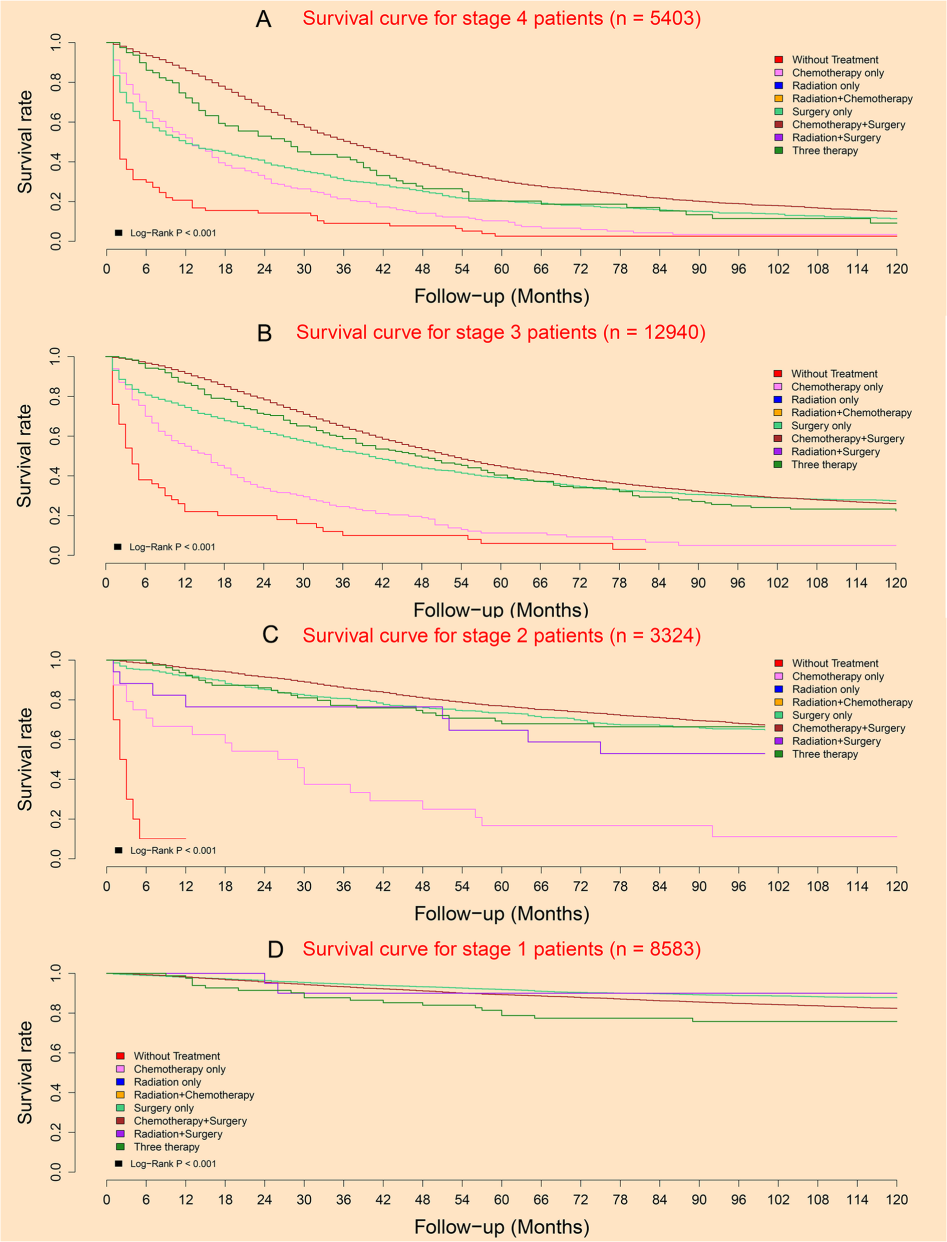
Subgroup survival curves under different treatments in different pathological stages: **A** Stage 4; **B** Stage 3; **C** Stage 2; **D** Stage 1

For patients with stage 4, 3 and 2, the survival of patients in chemotherapy plus surgery subgroup was the best, while the survival of patients in without treatment subgroup was the worst (Fig. 2A, B, and C). For patients with stage 1, there were four subgroups including surgery only subgroup, radiation plus surgery subgroup, chemotherapy plus surgery subgroup, and three therapy subgroup. Among these four treatments, the survival of three therapy subgroup was the worst, while the survival of surgery only subgroup and radiation plus surgery subgroup were better two (Fig. 2D).

### The 60-month survival rate and RMST at subgroup level

**Fig. 3:**
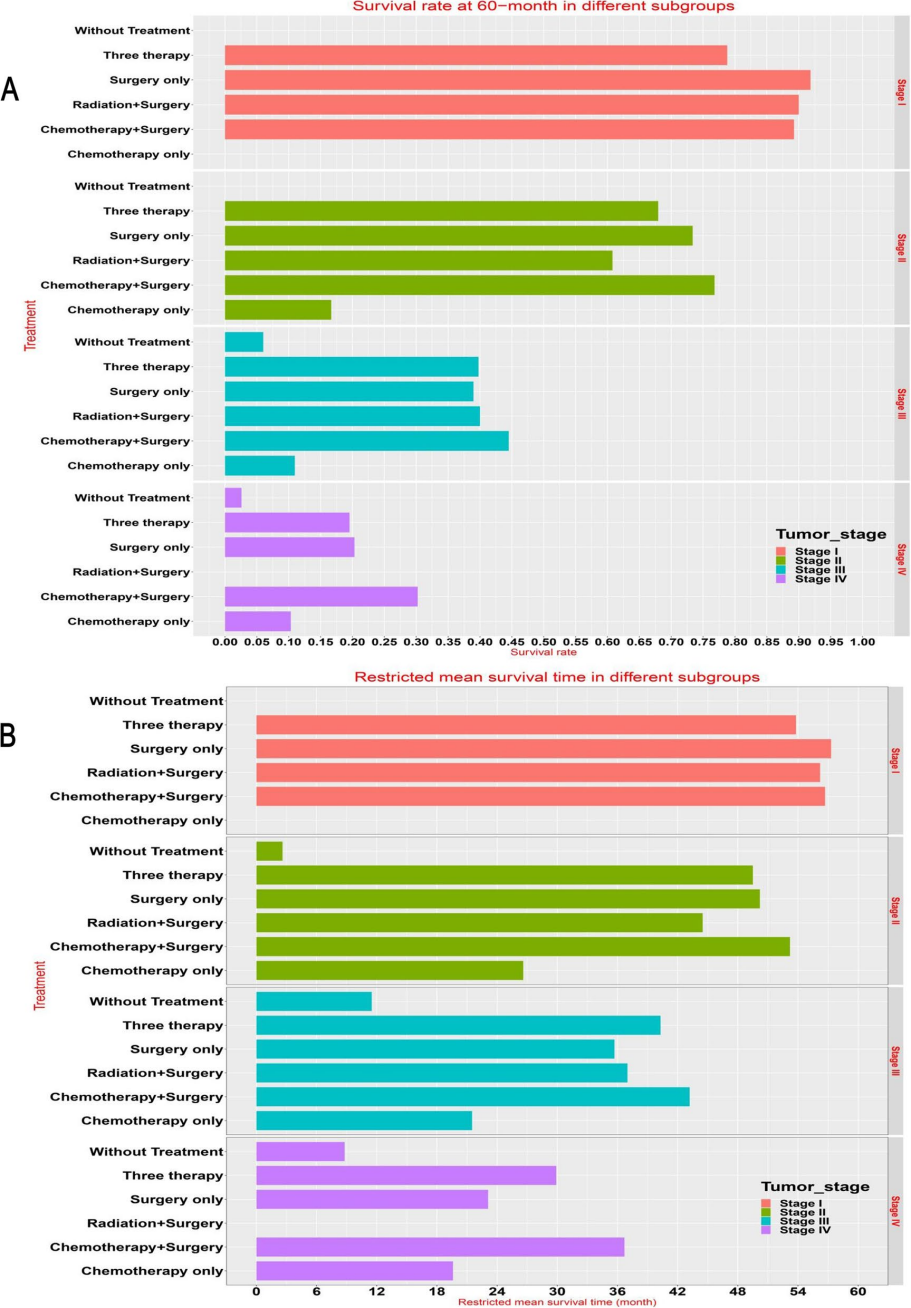
Subgroup survival rate (**A**) and restricted mean survival time (**B**) under different treatments in different pathological stages

**Fig. 4:**
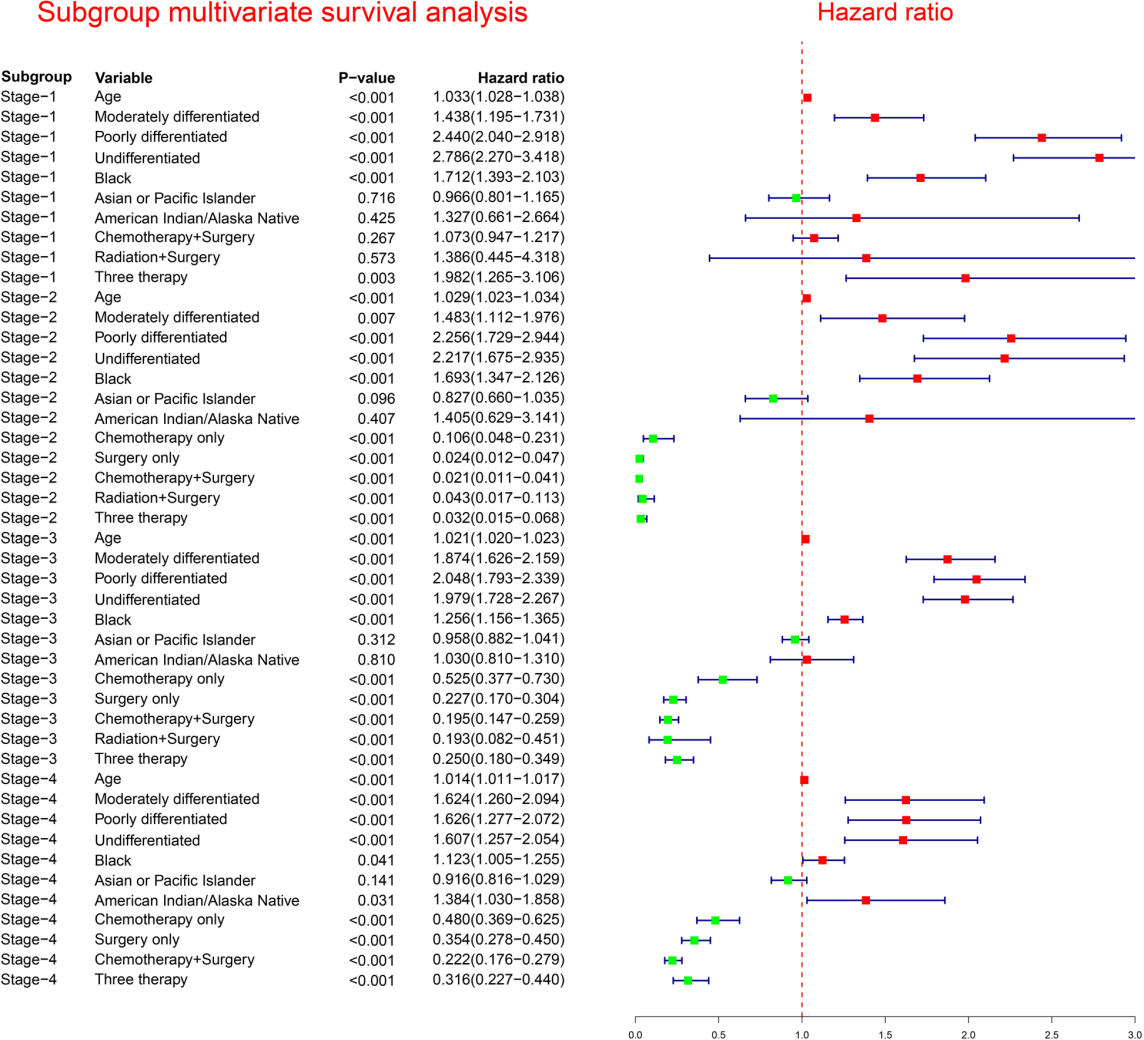
Subgroup multivariable survival analysis in different pathological stages

For patients with stage 4, 3 and 2, the 60-month Survival rate of patients in the chemotherapy plus surgery subgroup was the best (Fig. 3A and Supplementary document 4). For patients with stage 4, 3 and 2, restricted mean survival time of patients in chemotherapy plus surgery subgroup was the best (Fig. 4A and Supplementary document 4).

For patients with stage 1, the 60-month Survival rate of surgery only subgroup, chemotherapy plus surgery subgroup, and radiation plus surgery subgroup were superior to that of three therapy subgroup (Fig. 3B and Supplementary document 4). For patients with stage 1, restricted mean survival time of surgery only subgroup, chemotherapy plus surgery subgroup, and radiation plus surgery subgroup were superior to that of three therapy subgroup (Fig. 4B and Supplementary document 4).

### Multivariate Cox survival analysis at subgroup level

In multivariate analysis, the reference baseline subgroup was defined as white subgroup for race, well differentiated subgroup for grade, without treatment subgroup for treatment, Stage I subgroup for pathological stage. For patients with stage 1, multivariate Cox survival analysis indicated that the survival of surgery only subgroup, chemotherapy plus surgery subgroup, and radiation plus surgery subgroup were not significantly superior to that of three therapy subgroup after adjusting for confounding effects of age, grade and race (Fig. 4). For patients with stage 2 or stage 3, surgery only subgroup, chemotherapy only subgroup, chemotherapy plus surgery subgroup, three therapy subgroup, and radiation plus surgery subgroup were significantly superior to that of without treatment subgroup after adjusting for confounding effects of age, grade, and race (Fig. 4). For patients with stage 4, surgery only subgroup, chemotherapy only subgroup, chemotherapy plus surgery subgroup, and three therapy subgroup were significantly superior to that of without treatment subgroup after adjusting for confounding effects of age, grade, and race (Fig. 4).

### Prognostic model

Considering the accessibility and clinical generalization of indicators, the current study selected potential predictive indicators for the prognostic model from the following 12 clinical variables: age, stage, grade, PT, PN, PM, race, radiation, chemotherapy, surgery, laterality, and marital_status. The current study constructed a prognostic model for ovarian cancer patients based on 10 common clinical parameters including age, stage, grade, PT, PN, PM, race, radiation, chemotherapy, and surgery using accelerated failure time algorithm. The C indexes were 0.741 (95% confidence interval: 0.731–0.751) in model dataset and 0.738 (95% confidence interval: 0.726–0.750) in validation dataset. Brier score was 0.179 for model dataset and validation dataset.

### Bootstrap resampling dataset and internal validation

The bootstrap internal validation dataset (*n* = 30,255) was resampled from original dataset through boortrap resampling method (Supplementary document 5). The C indexes were 0.741 (95% confidence interval: 0.733–0.749) in bootstrap internal validation dataset. Brier score was 0.178 for bootstrap internal validation dataset.

### Individual mortality risk predictive system for PPPM

**Fig. 5:**
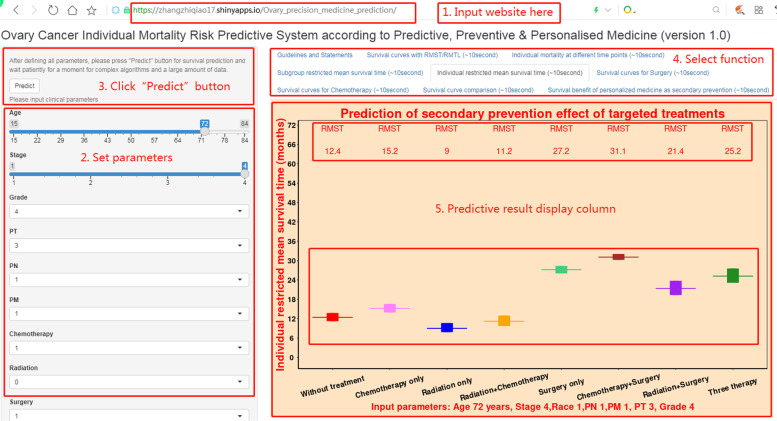
Introduction of operation for Individual Mortality Risk Predictive System

The current research developed an on-line individual mortality risk predictive system for PPPM. Figure 5 presented graphic description of operation and interpretation of current predictive system. This individual mortality risk predictive system was available at: https://zhangzhiqiao17.shinyapps.io/Ovary_precision_medicine_prediction/.

The individual mortality risk predictive system could provide individual predicted survival curve, individual restricted mean survival time, and individual predicted survival rate at a certain time point (Fig. 5). Next, the current study will show how to use this predictive system to predict the survival for a specific individual patient with age 72 years old, stage 4, Grade 4, PT 3, PN 1, PM 1, race white, radiation no, chemotherapy yes, and surgery yes.

### Individual predictive function for PPPM

**Fig. 6:**
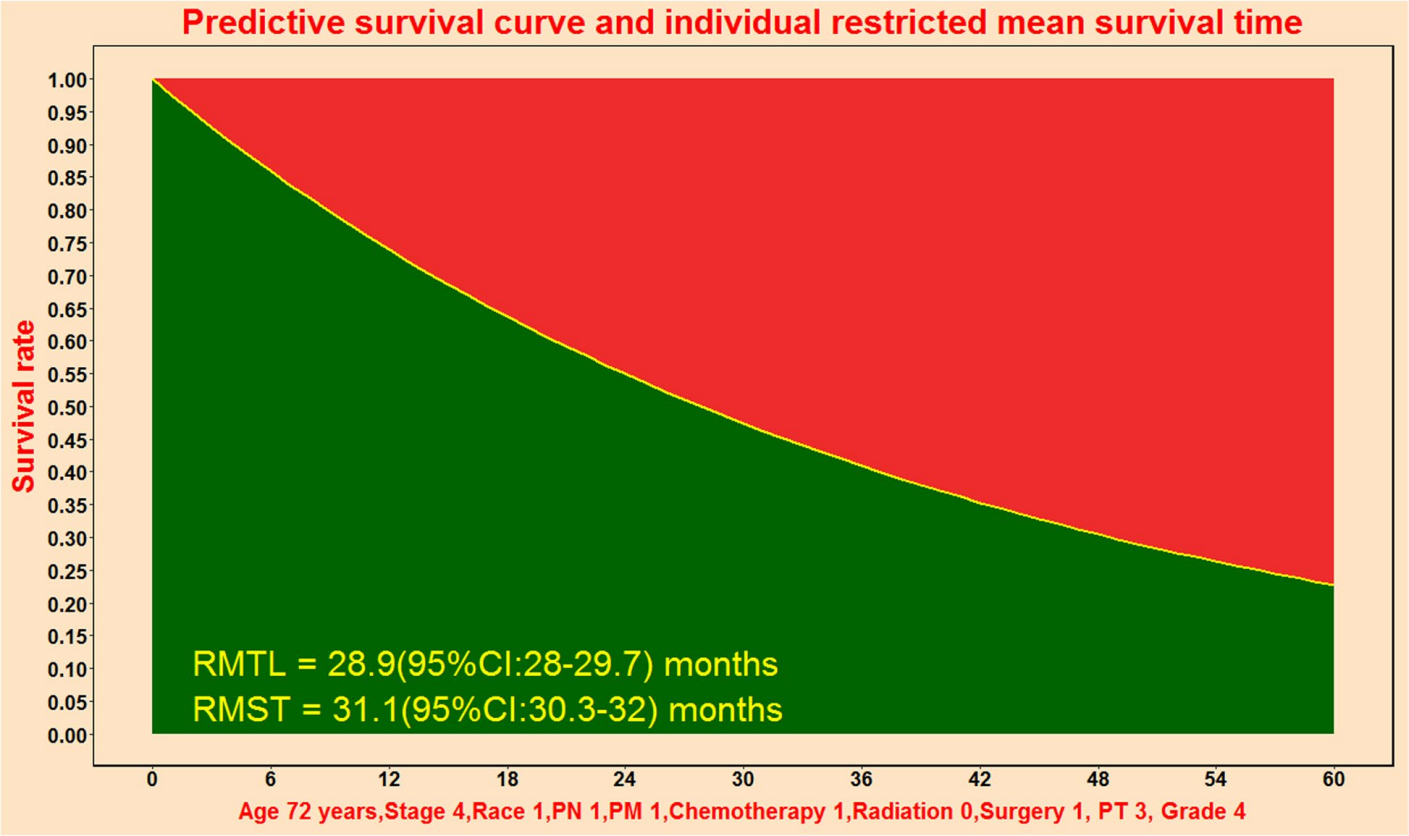
Predictive survival curve and individual restricted mean survival time

This predictive tool could generate individual predicted survival curve to perform individual predictive function for PPPM. The solid yellow line in Fig. 6 represented individual predicted survival curve for this individual patient. Restricted mean survival time was 31.1 months (green area) and restricted mean time loss was 28.9 months (red area).

### Risk stratification management function for PPPM

**Fig. 7:**
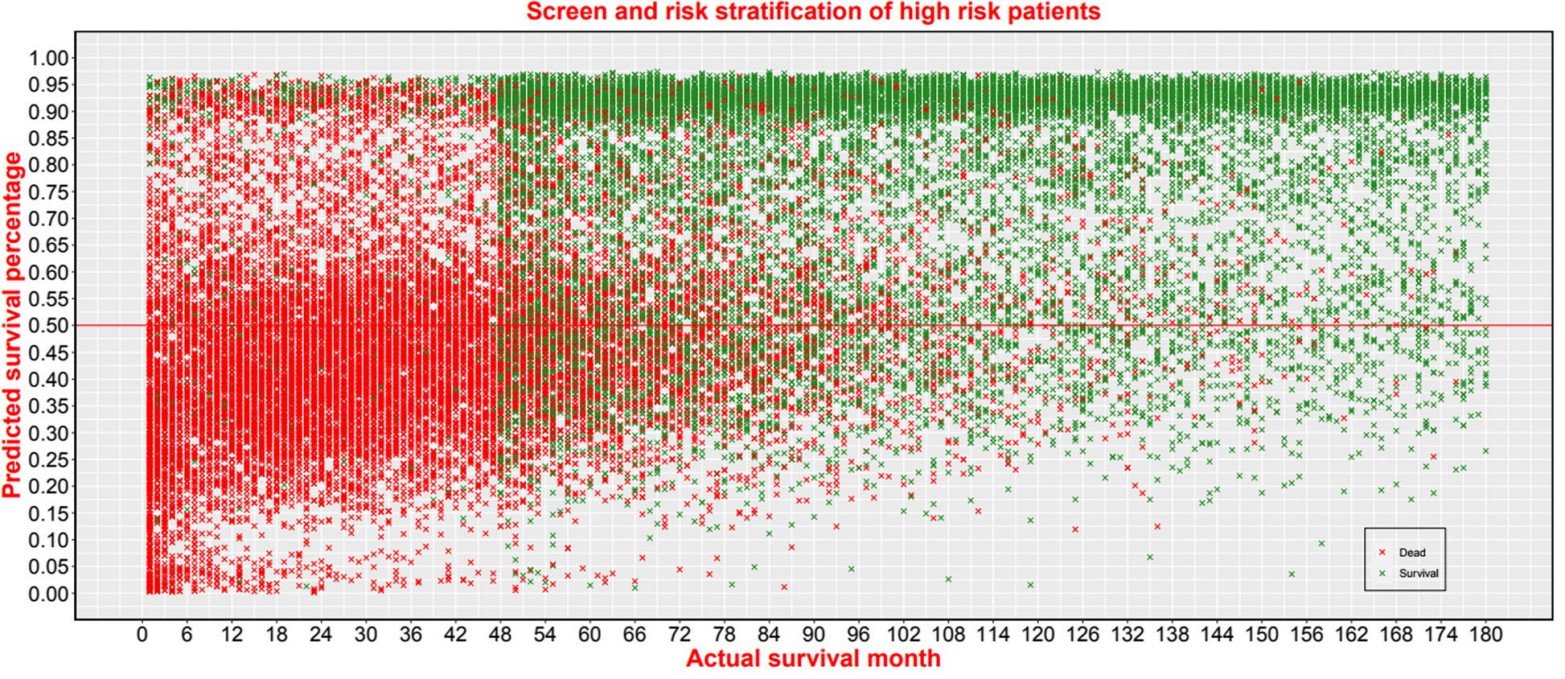
Scatter plot of actual survival time (X-axis) and predicted survival percentage (Y-axis)

To perform risk stratification management function for PPPM, the scatter plot was presented in Fig. 7. If we choose 0.5 as the boundary value between high risk patients and low risk patients, 12,257 (40.5%) patients out of total 30,255 patients were defined as high risk patients. In the high risk group (*n* = 12,257), 9,648 patients (76.8%) died during follow-up. Of the actual deaths (*n* = 15,672), 9648 patients were diagnosed as high risk patients with a sensitive rate of 61.6%.

**Fig. 8:**
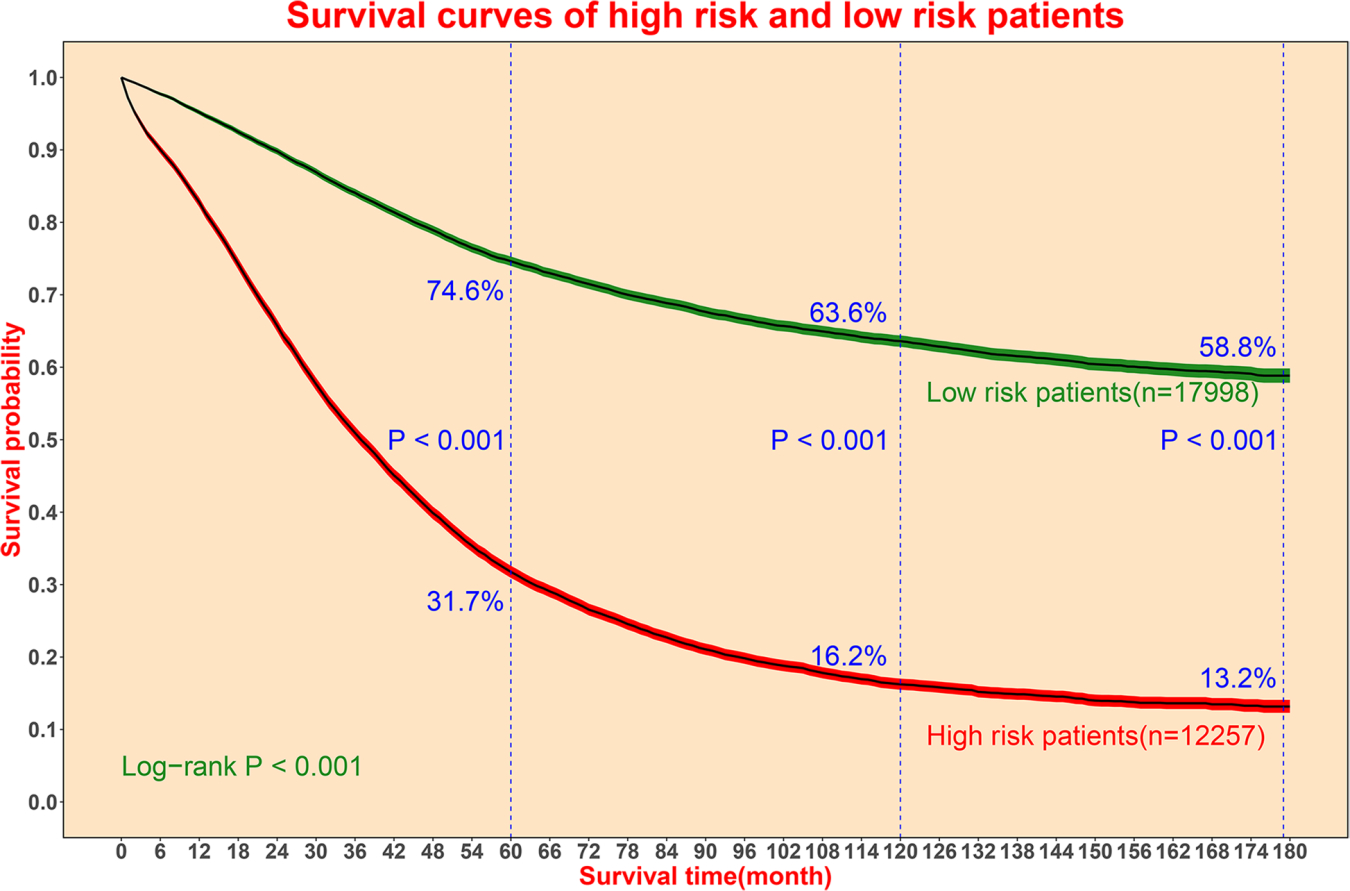
The survival curve analysis of high risk patients and low risk patients

Figure 8 showed the survival curves of patients in high risk group and low risk group. The 5-year and 10-year survival rate of high risk patients were 31.7% and 16.2%, respectively, which were significantly lower than those of low risk patients (74.6% and 63.6%).

### Prediction of secondary prevention effect of targeted treatment for PPPM

**Fig. 9:**
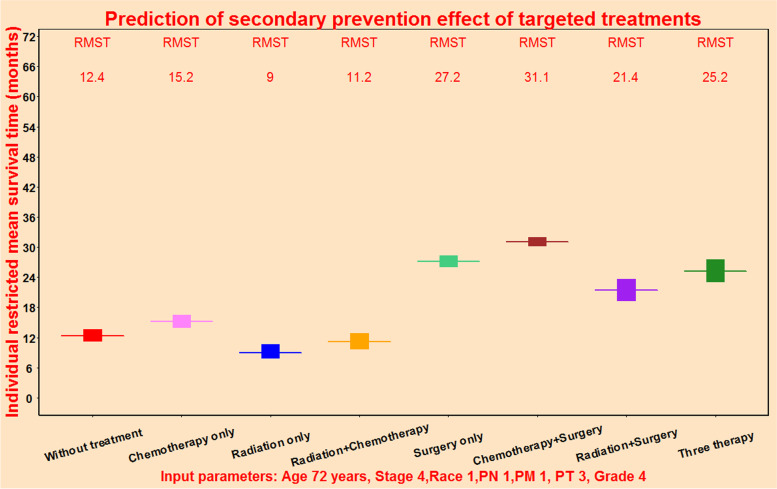
Survival benefit of different treatments as secondary prevention for an individual patient

The individual mortality risk predictive system could provide individual restricted mean survival time under different treatments for prediction of secondary prevention effect of targeted treatment for PPPM (Fig. 9). For this patient, the restricted mean survival time was estimated to be 27.2 months for patient receiving surgery only (light green block), and 31.1 months for patient receiving chemotherapy plus surgery treatment (brown block), indicating that chemotherapy plus surgery treatment could bring additional survival benefit of 3.9 months compared with receiving surgery only according to AFT algorithm. Through individual restricted mean survival time, we could compare the differences of survival benefits brought by eight treatments, so as to select the optimal treatment.

### Prediction of therapeutic survival benefit for PPPM

**Fig. 10:**
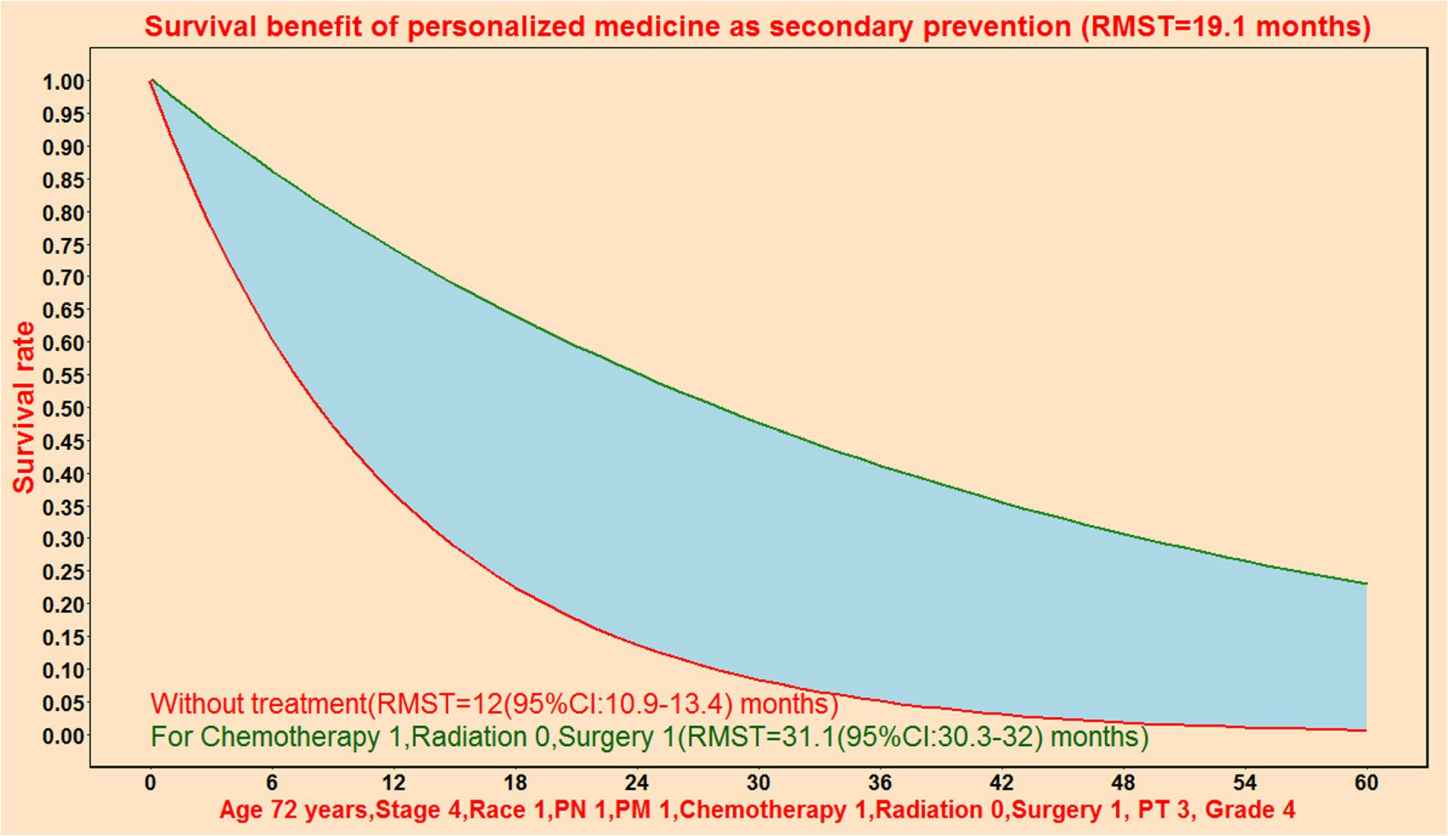
Comparison of prevention effect of two targeted treatment for an individual patient

The individual mortality risk predictive system could predict therapeutic survival benefit for PPPM (Fig. 10). As shown in Fig. 10, chemotherapy plus surgery treatment could bring additional survival benefit of 19.1 months compared without treatment according to Cox algorithm.

### Personalised medicine predictive function for PPPM

**Fig. 11:**
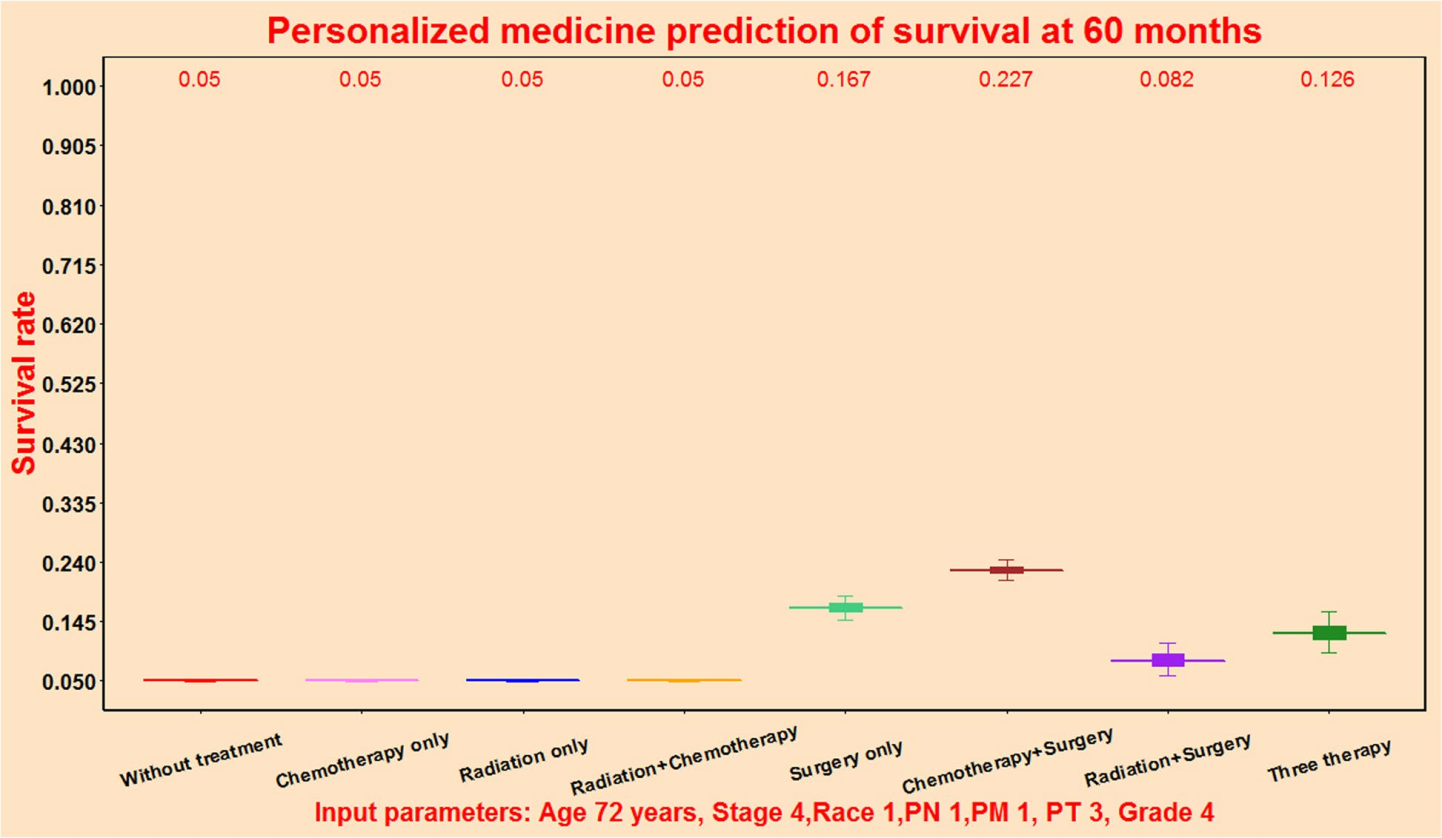
Personalised medicine prediction function of survival rate for an individual patient

This predictive tool could generate individual predicted survival rate at a certain time point to perform personalised medicine predictive function for PPPM. For this patient, the 60-month survival rate was estimated to be 16.7% for patient receiving surgery only (light green block), and 22.7% for patient receiving chemotherapy plus surgery treatment (brown block). This predictive function could display the predicted survival rate for a special individual patient under eight treatments at a specific time point, and help ovary cancer patient choose the best treatment (Fig. 11).

### Decision curve analysis

**Fig. 12:**
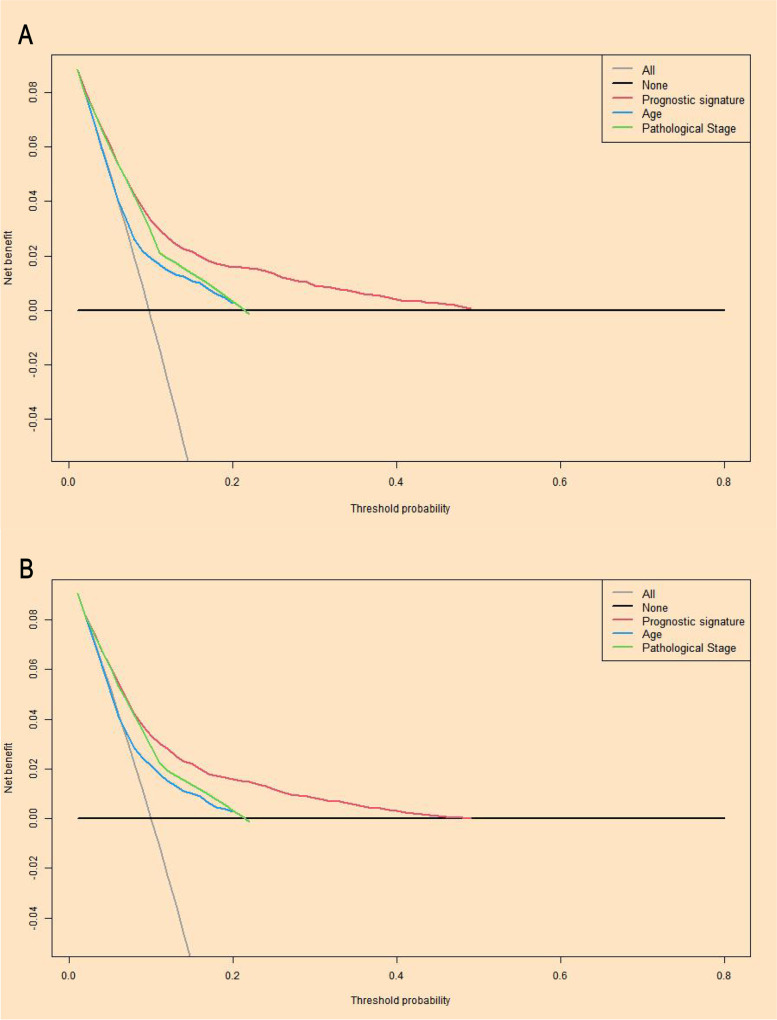
Decision curve analysis for model cohort (**A**) and validation cohort (**B**)

The current study used the Decision Curve Analysis method to verify the clinical utility value of different prognostic models. As shown in Fig. 12 A, the clinical predictive efficiency of the current prognostic model was superior to the traditional TMN pathological stage predictive system in model dataset. Figure 12B showed that the clinical predictive effectiveness of the prognostic model in the validation dataset was superior to the TMN pathological staging system.

## Discussion

The current study compared the survival benefits brought by various treatments in different stage ovarian cancer patients from three dimensions of overall level, subgroup level and individual level, demonstrating the differences of survival benefits of eight treatments in four stages. The current study constructed a prognosis model for ovarian cancer patients and developed an on-line application system to predict individual mortality risk.

The current study showed the difference of survival benefits brought by eight treatments through survival curve, predicted survival rate, restricted mean survival time, and hazard ratio at overall level. Subsequently, the current study explored the differences in the survival benefits of eight treatments in four stages. The comparison results provided a quantifiable reference standard for us to evaluate the differences of survival benefits brought by various treatments in four stages.

The restricted mean survival time at subgroup level has been applied to prognostic studies for different diseases. However, the current research proposed the concept of individual restricted mean survival time for the first time and successfully developed an on-line individual mortality risk predictive system. The individual mortality risk predictive system could provide individual predicted survival curve, individual restricted mean survival time, and individual restricted mean time loss. This individual predictive function could help us to explore the prognosis of ovary cancer patients at individual level.

Cox proportional hazard model is a semi-parametric predictive model, which needs to meet the proportional hazard hypothesis [31]. The lack of proportional hazard hypothesis will weaken the reliability of the prediction results [31]. The accelerated failure time model is a linear regression analysis model using log transformed linear model and log T as response variable in the case of censored survival data [32]. Accelerated failure time model is a valuable alternative to Cox model in survival analysis [32]. In the case of outliers or heavy-tailed errors, the robust loss function may be better than the traditional least square method in variable selection and prediction [33]. Compared with the Cox proportional hazard regression model, the accelerated failure time algorithm does not need to screen the predictive factors in advance, and the operation speed is faster [33]. The accelerated failure time model considers the statistical distribution of survival time and does not require to conform to the proportional hazard hypothesis, so it is mort suitable alternative to the Cox proportional hazard model for survival analysis [31].

Insufficients: First, although 30,255 ovarian cancer patients were enrolled in the current study, several subgroups still did’t obtain sufficient subjects. Future clinical studies with a larger sample size will help us deeply understand the differences in survival benefits of ovarian cancer patients in different subgroups. Second, the subjects in the current study were from 2004 to 2015, so the latest treatment information such as molecular targeted drugs was not recorded. In future research, it is necessary to incorporate the current mainstream treatment information into the research design, so as to expand the universality of the research results. Third, we searched several commonly used databases, including GEO database and TCGA database. However, due to the failure to find the dataset that meets sufficient survival information, detailed treatment information, complete pathological information and systematic follow-up information as the external validation dataset, the current study only carried out internal validation research. Independent external validation helps to further understand the effectiveness and clinical application value of current research conclusion. Fourth, several survival curves crossed in survival analysis chart. Considering that a portion of patients in the study cohort fall off during the follow-up period and resulted in right censoring for survival analysis, which may affect the performance of the subgroup survival curve, it is necessary to take into account the interference caused by the dropout patients in the study cohort when interpreting the performance of the survival curve.

In conclusion, the current study showed the differences of survival benefits of eight treatments in ovarian cancer patients with four different stages. The current study developed an on-line application system, which could provide individual predicted survival curve, individual restricted mean survival time, and individual predicted survival rate at a specific time point.

## Supplementary Information

**Additional file 1.** 

**Additional file 2.** 

## Data Availability

The study data is available at SEER database (https://seer.cancer.gov/).

## Abbreviations

RMST: Restricted mean survival time
RMTL: Restricted mean time loss
HR: Hazard ratio
CI: Confidence interval
AFT: Accelerated Failure Time
SEER: Surveillance Epidemiology and End Results
AJCC: American Joint Committee on Cancer
PPPM: Predictive, Preventive and Personalized Medicine
