# Supplementary material for: Differences of survival benefits brought by various treatments in ovarian cancer patients with different tumor stages
Source: J Ovarian Res. 2023 May 11;16:92. doi: 10.1186/s13048-023-01173-7 (PMC10176927; doi:10.1186/s13048-023-01173-7)
Supplement: Supplementary file 1 — Additional file 1. [file 13048_2023_1173_MOESM1_ESM.doc]

Supplementary document 1. The search strategy for eligible patients in SEER database

| Step | Search Code |
| --- | --- |
| First | {Site and Morphology.Diagnostic Confirmation} = ' Positive histology' |
| Second | {Age at Diagnosis.Age recode with <1 year olds} = '15-19 years','20-24 years','25-29 years','30-34 years','35-39 years','40-44 years','45-49 years','50-54 years','55-59 years','60-64 years','65-69 years','70-74 years','75-79 years','80-84 years' |
| Third | {Cause of Death (COD) and Follow-up.Survival months} != 'Unknown' |
| Fourth | {Cause of Death (COD) and Follow-up.Survival months flag} = 'Complete dates are available and there are more than 0 days of survival' |
| Fifth | {Cause of Death (COD) and Follow-up.SEER other cause of death classification} = 'Alive or dead due to cancer' |
| Sixth | {Other.Type of Reporting Source} != 'Autopsy only','Death certificate only' |
| Seventh | {Site and Morphology.Site recode ICD-O-3/WHO 2008} = 'Ovary' |

Supplementary Figure 2. Flow chart of patient enrollment


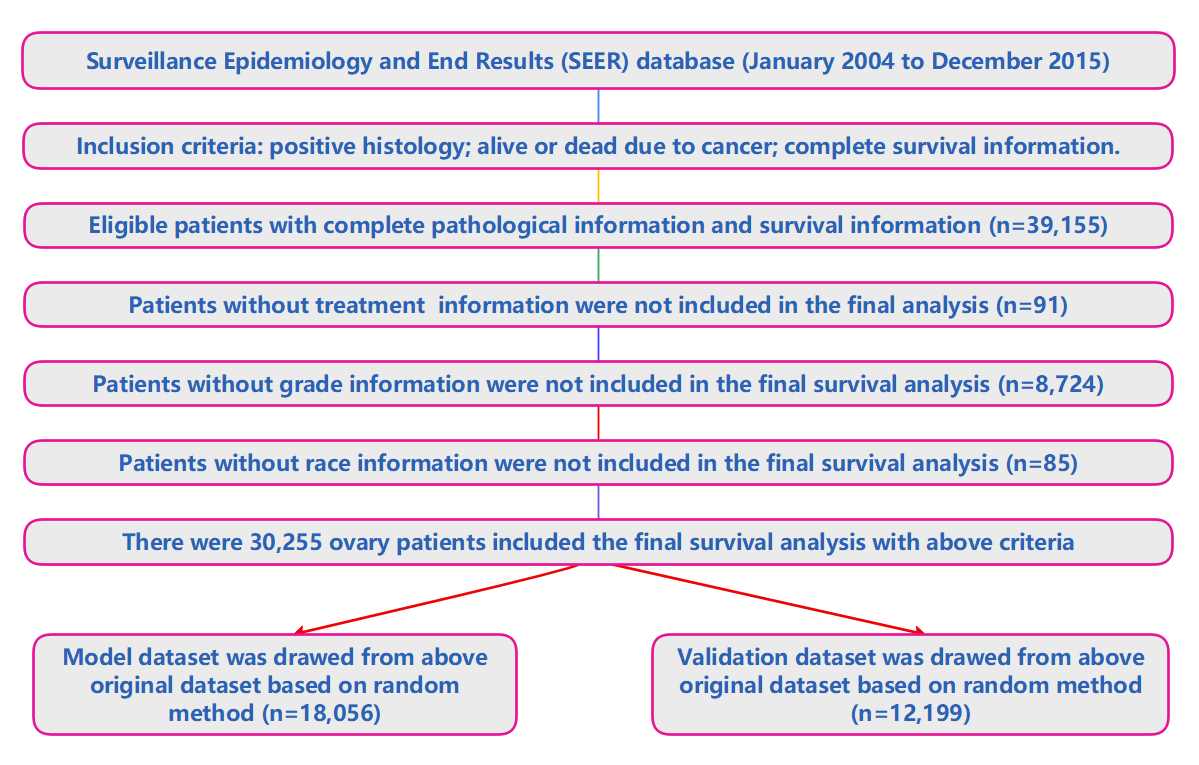


Supplementary document 3. Comparison analysis results between survival cohort and died cohort

| Parameter | Stratification | Total | Survival cohort | Died cohort | P value |
| --- | --- | --- | --- | --- | --- |
| Grade[n(%)] | Moderately differentiated | 5752 | 3593(11.88) | 2159(7.14) | <0.001 |
|  | Poorly differentiated | 13612 | 5122(16.93) | 8490(28.06) |  |
|  | Undifferentiated | 7364 | 2889(9.55) | 4475(14.79) |  |
|  | Well differentiated | 3527 | 2979(9.85) | 548(1.81) |  |
| Laterality[n(%)] | Bilateral, single primary | 12433 | 3877(12.81) | 8556(28.28) | <0.001 |
|  | Left | 8393 | 5263(17.4) | 3130(10.35) |  |
|  | Only one side - side unspecified | 145 | 42(0.14) | 103(0.34) |  |
|  | Paired site, no information concerning laterality | 746 | 162(0.54) | 584(1.93) |  |
|  | Right | 8538 | 5239(17.32) | 3299(10.9) |  |
| Race[n(%)] | American Indian/Alaska Native | 220 | 93(0.31) | 127(0.42) | <0.001 |
|  | Asian or Pacific Islander | 2814 | 1675(5.54) | 1139(3.76) |  |
|  | Black | 1893 | 760(2.51) | 1133(3.74) |  |
|  | White | 25328 | 12055(39.84) | 13273(43.87) |  |
| Stage[n(%)] | Stage I | 8583 | 7411(24.5) | 1172(3.87) | <0.001 |
|  | Stage II | 3325 | 2207(7.29) | 1118(3.7) |  |
|  | Stage III | 12940 | 3972(13.13) | 8968(29.64) |  |
|  | Stage IV | 5407 | 993(3.28) | 4414(14.59) |  |
| PT[n(%)] | T1 | 9128 | 7696(25.44) | 1432(4.73) | <0.001 |
|  | T2 | 4387 | 2614(8.64) | 1773(5.86) |  |
|  | T3 | 16740 | 4273(14.12) | 12467(41.21) |  |
| PN[n(%)] | No | 23238 | 12584(41.59) | 10654(35.21) | <0.001 |
|  | Yes | 7017 | 1999(6.61) | 5018(16.59) |  |
| PM[n(%)] | No | 24848 | 13590(44.92) | 11258(37.21) | <0.001 |
|  | Yes | 5407 | 993(3.28) | 4414(14.59) |  |
| Radiation_regimen[n(%)] | Beam radiation | 372 | 137(0.45) | 235(0.78) | <0.001 |
|  | Combination of beam with implants or isotopes | 28 | 15(0.05) | 13(0.04) |  |
|  | No radiation | 29771 | 14385(47.55) | 15386(50.85) |  |
|  | Radiation, method or source not specified | 18 | 4(0.01) | 14(0.05) |  |
|  | Radioactive implants (includes brachytherapy) | 60 | 39(0.13) | 21(0.07) |  |
|  | Radioisotopes | 6 | 3(0.01) | 3(0.01) |  |
| Sequential_therapy[n(%)] | Intraoperative radiation before/after surgery | 2 | 1(0.0) | 1(0.0) | 0.008 |
|  | Intraoperative radiation | 2 | 0(0.0) | 2(0.01) |  |
|  | No radiation and/or cancer-directed surgery | 29786 | 14387(47.55) | 15399(50.9) |  |
|  | Radiation after surgery | 433 | 189(0.62) | 244(0.81) |  |
|  | Radiation before and after surgery | 4 | 0(0.0) | 4(0.01) |  |
|  | Radiation prior to surgery | 21 | 5(0.02) | 16(0.05) |  |
|  | Sequence unknown, but both were given | 7 | 1(0.0) | 6(0.02) |  |
| Surgery[n(%)] | No | 567 | 50(0.17) | 517(1.71) | <0.001 |
|  | Yes | 29688 | 14533(48.04) | 15155(50.09) |  |
| Chemotherapy[n(%)] | No | 7009 | 4461(14.74) | 2548(8.42) | <0.001 |
|  | Yes | 23246 | 10122(33.46) | 13124(43.38) |  |
| Radiation[n(%)] | No | 29771 | 14385(47.55) | 15386(50.85) | <0.001 |
|  | Yes | 484 | 198(0.65) | 286(0.95) |  |
| Site_recode[n(%)] | Clear cell adenocarcinoma | 1734 | 1131(3.74) | 603(1.99) | <0.001 |
|  | Cystadenocarcinoma | 17368 | 6370(21.05) | 10998(36.35) |  |
|  | Dysgerminoma | 23 | 21(0.07) | 2(0.01) |  |
|  | Endometrioid | 4313 | 3366(11.13) | 947(3.13) |  |
|  | Mixed cell adenocarcinoma | 1807 | 1010(3.34) | 797(2.63) |  |
|  | Mixed germ cell | 46 | 44(0.15) | 2(0.01) |  |
|  | Mucinous adenocarcinoma | 1354 | 975(3.22) | 379(1.25) |  |
|  | Other carcinoma | 2955 | 1070(3.54) | 1885(6.23) |  |
|  | Sex cord and other specialized gonadal | 242 | 201(0.66) | 41(0.14) |  |
|  | Teratoma | 389 | 376(1.24) | 13(0.04) |  |
|  | Yolk sac | 24 | 19(0.06) | 5(0.02) |  |
| Histology_recode[n(%)] | adenomas and adenocarcinomas | 9203 | 5947(19.66) | 3256(10.76) | <0.001 |
|  | complex epithelial neoplasms | 120 | 86(0.28) | 34(0.11) |  |
|  | complex mixed and stromal neoplasms | 409 | 111(0.37) | 298(0.98) |  |
|  | cystic, mucinous and serous neoplasms | 18743 | 7349(24.29) | 11394(37.66) |  |
|  | ductal and lobular neoplasms | 1 | 1(0.0) | 0(0.0) |  |
|  | epithelial neoplasms, NOS | 695 | 257(0.85) | 438(1.45) |  |
|  | germ cell neoplasms | 496 | 470(1.55) | 26(0.09) |  |
|  | mucoepidermoid neoplasms | 1 | 0(0.0) | 1(0.0) |  |
|  | specialized gonadal neoplasms | 242 | 201(0.66) | 41(0.14) |  |
|  | squamous cell neoplasms | 196 | 78(0.26) | 118(0.39) |  |
|  | transitional cell papillomas and carcinomas | 123 | 74(0.24) | 49(0.16) |  |
|  | unspecified neoplasms | 26 | 9(0.03) | 17(0.06) |  |

Note: RMST: restricted mean survival time.

Supplementary document 4. Subgroup analysis results for different subgroups

| Treatment | Tumor_stage | Number | Age | RMST | Median_survival | Survival_rate |
| --- | --- | --- | --- | --- | --- | --- |
| Surgery only | Stage I | 3909 | 52.1 | 57.3 | NA | 0.918 |
| Chemotherapy+Surgery | Stage I | 4557 | 53.2 | 56.7 | NA | 0.893 |
| Radiation+Surgery | Stage I | 21 | 50.9 | 56.2 | NA | 0.900 |
| Three therapy | Stage I | 82 | 52.2 | 53.8 | NA | 0.788 |
| Chemotherapy only | Stage II | 24 | 67.4 | 26.6 | 27.5 | 0.167 |
| Surgery only | Stage II | 639 | 59.7 | 50.2 | NA | 0.734 |
| Chemotherapy+Surgery | Stage II | 2553 | 57.8 | 53.2 | NA | 0.768 |
| Radiation+Surgery | Stage II | 17 | 61.7 | 44.5 | NA | 0.608 |
| Three therapy | Stage II | 80 | 53 | 49.5 | NA | 0.680 |
| Without Treatment | Stage III | 50 | 69.7 | 11.5 | 4 | 0.060 |
| Chemotherapy only | Stage III | 147 | 69.4 | 21.5 | 16 | 0.110 |
| Surgery only | Stage III | 1576 | 62.7 | 35.7 | 40 | 0.390 |
| Chemotherapy+Surgery | Stage III | 10981 | 59.8 | 43.2 | 53 | 0.445 |
| Radiation+Surgery | Stage III | 10 | 59 | 37 | 46 | 0.400 |
| Three therapy | Stage III | 173 | 57.2 | 40.3 | 48 | 0.398 |
| Without Treatment | Stage IV | 79 | 67.4 | 8.8 | 2 | 0.026 |
| Chemotherapy only | Stage IV | 228 | 65.7 | 19.6 | 13 | 0.103 |
| Surgery only | Stage IV | 679 | 64.2 | 23.1 | 12 | 0.203 |
| Chemotherapy+Surgery | Stage IV | 4325 | 60.4 | 36.7 | 37 | 0.302 |
| Three therapy | Stage IV | 79 | 54.6 | 29.9 | 28 | 0.195 |
| Without Treatment | all_Stage | 146 | 67.8 | 10.9 | 3 | 0.060 |
| Chemotherapy only | all_Stage | 406 | 67.1 | 21.3 | 15 | 0.115 |
| Radiation+Chemotherapy | all_Stage | 10 | 59.8 | 25.6 | 19.5 | 0.150 |
| Surgery only | all_Stage | 6803 | 56.4 | 48.2 |  | 0.706 |
| Chemotherapy+Surgery | all_Stage | 22416 | 58.3 | 45.8 | 72 | 0.544 |
| Radiation+Surgery | all_Stage | 55 | 58.0 | 45.1 | 175 | 0.650 |
| Three therapy | all_Stage | 414 | 54.9 | 42.9 | 60 | 0.496 |

Note: RMST: restricted mean survival time.
